# RETRACTION: Perceived Stigmatisation and Reliability of Questionnaire in the Survivors With Burns Wound: A Systematic Review and Meta‐Analysis

**DOI:** 10.1111/iwj.70257

**Published:** 2025-02-25

## Abstract

**RETRACTION:** FarzanR. , HosseiniS. J. , FiroozM. , TabarianM. S. , JamshidbeigiA. , SamidoustP. , SarafiM. , MahdiabadiM. Z. , VajargahP. G. , MollaeiA. , KarkhahS. , TakasiP. , ParviziA. , HaddadiS. , “Perceived Stigmatisation and Reliability of Questionnaire in the Survivors With Burns Wound: A Systematic Review and Meta‐Analysis,” International Wound Journal 20, no. 8 (2023): 3391–3403, 10.1111/iwj.14176.37016493 PMC10502297

The above article, published online on 04 April 2023, in Wiley Online Library (http://onlinelibrary.wiley.com/), has been retracted by agreement between the journal Editor in Chief, Professor Keith Harding; and John Wiley & Sons Ltd. A third party reported to the journal that they had found evidence of excessive self‐citations in the reference list of this article. The publisher confirmed multiple instances of redundant self‐citations. Upon further investigation, the publisher also concluded that this article was accepted solely on the basis of a compromised peer review process. In view of the clear evidence of compromised peer review, the parties agreed that the paper must be retracted. The authors disagree with the retraction.

**RETRACTION:** R. Farzan , S. J. Hosseini , M. Firooz , M. S. Tabarian , A. Jamshidbeigi , P. Samidoust , M. Sarafi , M. Z. Mahdiabadi , P. G. Vajargah , A. Mollaei , S. Karkhah , P. Takasi , A. Parvizi , S. Haddadi , “Perceived Stigmatisation and Reliability of Questionnaire in the Survivors With Burns Wound: A Systematic Review and Meta‐Analysis,” International Wound Journal 20, no. 8 (2023): 3391–3403, 10.1111/iwj.14176.37016493 PMC10502297

The above article, published online on 04 April 2023, in Wiley Online Library (http://onlinelibrary.wiley.com/), has been retracted by agreement between the journal Editor in Chief, Professor Keith Harding; and John Wiley & Sons Ltd. A third party reported to the journal that they had found evidence of excessive self‐citations in the reference list of this article. The publisher confirmed multiple instances of redundant self‐citations. Upon further investigation, the publisher also concluded that this article was accepted solely on the basis of a compromised peer review process. In view of the clear evidence of compromised peer review, the parties agreed that the paper must be retracted. The authors disagree with the retraction.

